# The relationship between addiction to smartphone usage and depression among adults: a cross sectional study

**DOI:** 10.1186/s12888-018-1745-4

**Published:** 2018-05-25

**Authors:** Aljohara A. Alhassan, Ethar M. Alqadhib, Nada W. Taha, Raneem A. Alahmari, Mahmoud Salam, Adel F. Almutairi

**Affiliations:** 1 College of Rehabilitation and Health Sciences, Princess Noura Bint Abdulrahman University, Riyadh, Saudi Arabia; 2 King Abdullah International Medical Research Center, King Saud bin Abdulaziz University for Health Sciences, College of Applied Medical Sciences, Echography Cardiovascular Technology, Ministry of National Guard - Health Affairs, Riyadh, Saudi Arabia; 3 King Abdullah International Medical Research Center, King Saud bin Abdulaziz University for Health Sciences, Science and Technology Unit, Ministry of National Guard - Health Affairs, Riyadh, 11426 Saudi Arabia

**Keywords:** Smartphone addiction, Depression, Age, Education, Middle East

## Abstract

**Background:**

Addiction to smartphone usage is a common worldwide problem among adults, which might negatively affect their wellbeing. This study investigated the prevalence and factors associated with smartphone addiction and depression among a Middle Eastern population.

**Methods:**

This cross-sectional study was conducted in 2017 using a web-based questionnaire distributed via social media. Responses to the Smartphone Addiction Scale - Short version (10-items) were rated on a 6-point Likert scale, and their percentage mean score (PMS) was commuted. Responses to Beck’s Depression Inventory (20-items) were summated (range 0–60); their mean score (MS) was commuted and categorized. Higher scores indicated higher levels of addiction and depression. Factors associated with these outcomes were identified using descriptive and regression analyses. Statistical significance was set at *P* < 0.05.

**Results:**

Complete questionnaires were 935/1120 (83.5%), of which 619 (66.2%) were females and 316 (33.8%) were males. The mean ± standard deviation of their age was 31.7 ± 11  years. Majority of participants obtained university education 766 (81.9%), while 169 (18.1%) had school education. The PMS of addiction was 50.2 ± 20.3, and MS of depression was 13.6 ± 10.0. A significant positive linear relationship was present between smart phone addiction and depression (y = 39.2 + 0.8×; *P* < 0.001). Significantly higher smartphone addiction scores were associated with younger age users, (β = − 0.203, adj. *P* = 0.004). Factors associated with higher depression scores were school educated users (β = − 2.03, adj. *P* = 0.01) compared to the university educated group and users with higher smart phone addiction scores (β =0.194, adj. *P* < 0.001).

**Conclusions:**

The positive correlation between smartphone addiction and depression is alarming. Reasonable usage of smart phones is advised, especially among younger adults and less educated users who could be at higher risk of depression.

**Electronic supplementary material:**

The online version of this article (10.1186/s12888-018-1745-4) contains supplementary material, which is available to authorized users.

## Background

Addiction to smartphone usage is a common problem among adults worldwide. It manifests itself in the excessive usage of their phones, while engaged in other activities such as studying, driving, social gatherings and even sleeping [1]. However, many people fail to realize that addiction to smartphone usage is a serious issue that can have a negative effect on the person’s thoughts, behavior, tendencies, feelings, and sense of well-being [2]. In particular, it can be a risk factor for depression, loneliness, anxiety and sleep disturbances [3]. As per the Mental Health Foundation in the United Kingdom, people with depression experience an unhappy mood, loss of interest or pleasure, feelings of guilt or low self-worth, disturbed sleep or appetite, low energy, and poor concentration. Depressive and anxiety disorders are two main common disorders that are highly prevalent globally, as over 300 million people are estimated to suffer from depression, which is equivalent to 4.4% of the world’s population [4]. It is speculated that not only addiction to smartphone usage can affect one’s mental and behavioral status, but also that those with mood disorders are more likely to become addicted to using their smartphones [5].

Numerous tools have been utilized in literature to assess the same phenomenon, but with different terms such as excessive smart phone usage, smartphone addiction, dependency on smart phones, internet addiction, problematic mobile phone usage, and so on. Remarkably, there was a tendency to use a non-pathological terminology, such as “Problematic Smartphone Use,” rather than the term smartphone addiction [6]. Addiction manifests itself in various forms such as preoccupation, tolerance, lack of control, withdrawal, mood modification, conflict, lies, excessive use and loss of interest [7]. Several studies have found that women are more likely to develop an addiction to smartphone usage than men [8]. This was viewed as a positive way for people to stay connected in social relationships. One study clarified that women like to show affection to their families using their smartphones while men use phones for efficiency and practicality [3, 8]. Though there are several studies on this topic, no study has proven this connection so far. Smartphone addiction has been found to be correlated with various physical and psychological issues, as indicated in a number of studies that tested this relationship among various age groups. For example, one study found that people with depression, social anxiety and loneliness had different uses for their smartphones compared to others [9]. People with social anxiety made fewer outgoing calls, as well as, fewer text messages than those without social anxiety [9]. It was reported that high levels of smartphone addiction were correlated with low self-esteem, loneliness, depression and shyness [10].

Depression is a general reflection of the psychological wellbeing that is thought to be highly correlated with addiction to smartphone usage. The majority of studies on this issue revealed that there is a relationship between these two variables; however, all these studies were conducted in specific populations. In Turkey, females were more likely to develop an addiction to smartphone usage compared to males [11], while in Austria chronic stress, low emotional stability, female gender, young age and depression were associated with problematic mobile usage [12]. In Taiwan, adolescents with significant depression were more likely to have four or more symptoms of problematic cell phone usage [13]. Depression and anxiety disorders were reported around 18% among adults in Saudi Arabia [14]. Each community is unique in terms of its norms, practices, behaviors and traditions. Even within a single Middle Eastern setting, ethno-national affiliations may influence how people associate between insights and self-stigma [15]. Those residing in the Middle Eastern region consist of heterogeneous systems of social differentiation based on ethnic, linguistic, sectarian, familial, tribal, regional, socioeconomic, and national identities. Ethnic Arab people likewise follow more than one faith tradition. It is worth mentioning that Western cultural norms have been disseminated into the Arab world, but this effect has been experienced differently in communities and across societies [16]. The aim of this study was to investigate the relationship between addiction to smartphone usage and self-reported depression among a Middle Eastern population to better understand this relationship in such a region. This was fulfilled by presenting the prevalence of smart phone addiction and depression, describing the sample characteristics and identifying factors associated with both smartphone addiction and depression.

## Methods

A cross-sectional study was conducted in 2017 by distributing a pre-validated web-based questionnaire through common social media networks, among the Saudi Arabian population. The usage of social networking channels guaranteed maximum distribution throughout various geographical regions and boosted the representativeness of the sample. A brief description of the study objectives was presented, and by convenience, those who agreed to respond to the questionnaire confirmed their agreement electronically. Incomplete questionnaires were dropped out. Participants were requested to report their nationality, age and region upon submission. Participants below 18 years of age were excluded. The data retrieved were stored in a private institutional server. This study was approved by the Institutional Review Board of the Saudi Ministry of National Guard Health Affairs (MNG-HA), Riyadh, Saudi Arabia (Rss 17/003). Participation was voluntary with assurance about the confidentiality of their information, as no identifiers or personal information were collected.

The data collection tool comprised of three sections, namely the participants’ characteristics, the smart phone addiction assessment tool and the self-reported depression scale. These tools have been widely used in literature [11, 17]. Participants’ characteristics were nationality, sex, age (years), level of education (school vs. university level), place of residence (region) and financial income level (<$1500 vs. ≥$1500). Participants with school educational level were those who stopped their education prior enrollment in university, while those with university educational levels were those either currently enrolled or have graduated from university. The level of smartphone addiction was assessed using the Smartphone Addiction Scale-Short Version (SAS-SV), which is a validated scale developed from the original Smartphone Addiction Scale (SAS) by Kwon 2013. It is a self-reported scale containing 10-items, each rated on a 6- point Likert scale from one “strongly disagree” to six “strongly agree”. The total scores were summated, then converted to percentage mean scores (PMS), where higher scores indicated higher levels of smartphone addiction. In the original scale of SAS, the cut-off point was not reported [18]. A cutoff point at of 31/60 in males and 33/60 in females was utilized in the short version SAS by one Spanish-Belgium study [19]. Since no gender differences were reported between these cut off points [19], a cut-off point of score 32/60 or PMS = 53.3% was accounted in this study with subjects having PMS > 53% classified as excessive users or probably addicts. The concurrent validity and internal consistency were obtained for SAS-SV (Cronbach’s alpha: 0.91) [18].

The Beck’s Depression Inventory (BDI II) second edition is a tool used to assess the severity of depressive symptoms and to objectively quantify depression signs, but it’s not a diagnostic tool. The BDI II consists of 20 items rated on a 4-point scale. The total score ranges from 0 to 60, as higher scores indicated higher levels of depression. Participants with scores 0–9 were classified as “having ups and downs but considered normal”, 10–15 classified as “having mild mood disturbance”, 16–19 classified as “borderline clinical depression”, 20–29 classified as “having moderate depression”, 30–39 classified with “severe depression” and ≥ 40 as having “extreme depression”. This tool showed a Cronbach’s alpha reliability coefficient of 0.89 [20]. The Arabic version that was used in this study was previously translated by another researcher with a coefficients alpha of 0.82 [21].

Data were analyzed using SPSS; IBM (version 24). Descriptive statistics including mean, standard deviation, frequency and percentages were used to present the participants’ demographics and outcomes. The smart phone addiction and self-reported depression scores were analyzed as continuous variables and categorized qualitatively. Independent student t- test and one way ANOVA were used for bivariate analysis. Pearson’s correlation was used to determine the nature and significance of relationship between the two outcome measures. Factors significantly associated with smart phone addiction and depression were presented after controlling for any possible confounder using two linear regression models.

## Results

### Sample characteristics

Response rate was 935/1120 (83.5%). The younger age group (18–35 years) were 609 (65.1%), followed by middle age group (36–54 years) 303 (32.4%) and older age group (≥55 years) 23 (2.5%), with a mean of age ± standard deviation 31.7 ± 11 years. Catchment areas varied between all regions of the Kingdom, with highest response in Central region 668 (71.5%) and least in Northern region 5(0.6%). Females comprised 619 (66.2%) and males 16 (33.8%) of the sample. The income of 418 (44.7%) was <$1300, while 517 (55.3%) was ≥$1300. Majority of sample 766 (81.9%) were university educated, while 169 (18.1%) were school educated, Table 1.

**Table 1:** Description of sample characteristics

	*N* = 935 (100%)
Age (years)	
Young (18–35)	609 (65.1)
Middle (36–54)	303 (32.4)
Old (≥55)	23 (2.5)
x ± SD	31.7 ± 10.98
Gender	
Male	316 (33.8)
Female	619 (66.2)
Income	
Less than $1300	418 (44.7)
Between $1300 & $3700	316 (33.8)
More than $3700	201 (21.5)
Educational level	
School	169 (18.1)
University	766 (81.9)
Regional distribution	
North	5 (0.6)
South	47 (5.0)
Eastern	74 (7.9)
Western	121 (12.9)
Central	668 (71.5)
Others	20 (2.1)

*Abbreviations*: *x* mean, *SD* standard deviation

### Smartphone addiction and Beck depression

**Fig. 1:**
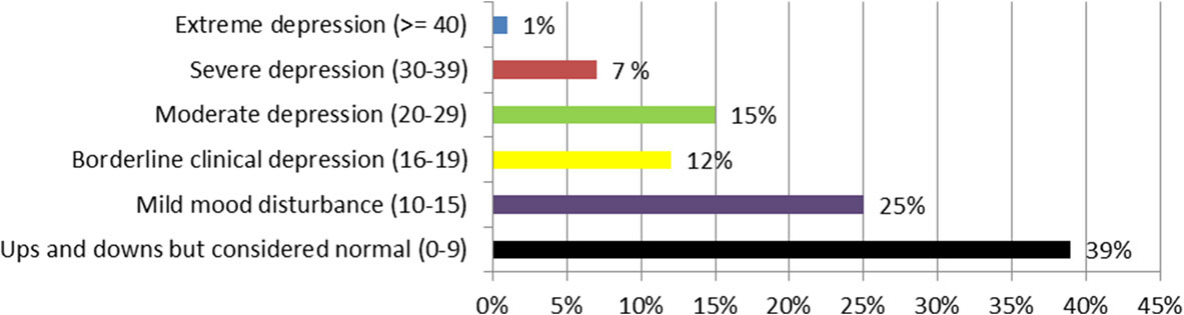
Various levels of depression

**Fig. 2:**
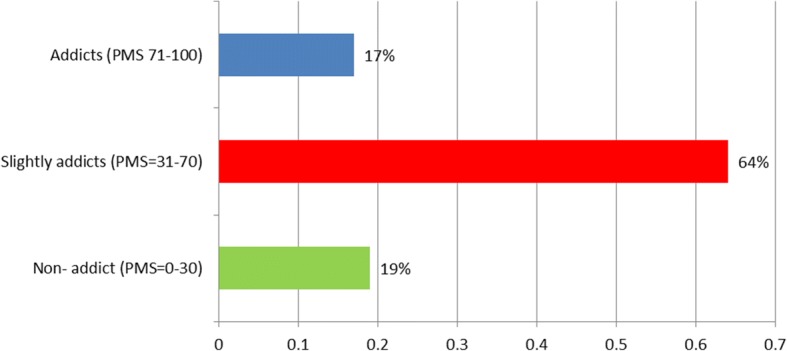
Various levels of smart phone addiction

**Fig. 3:**
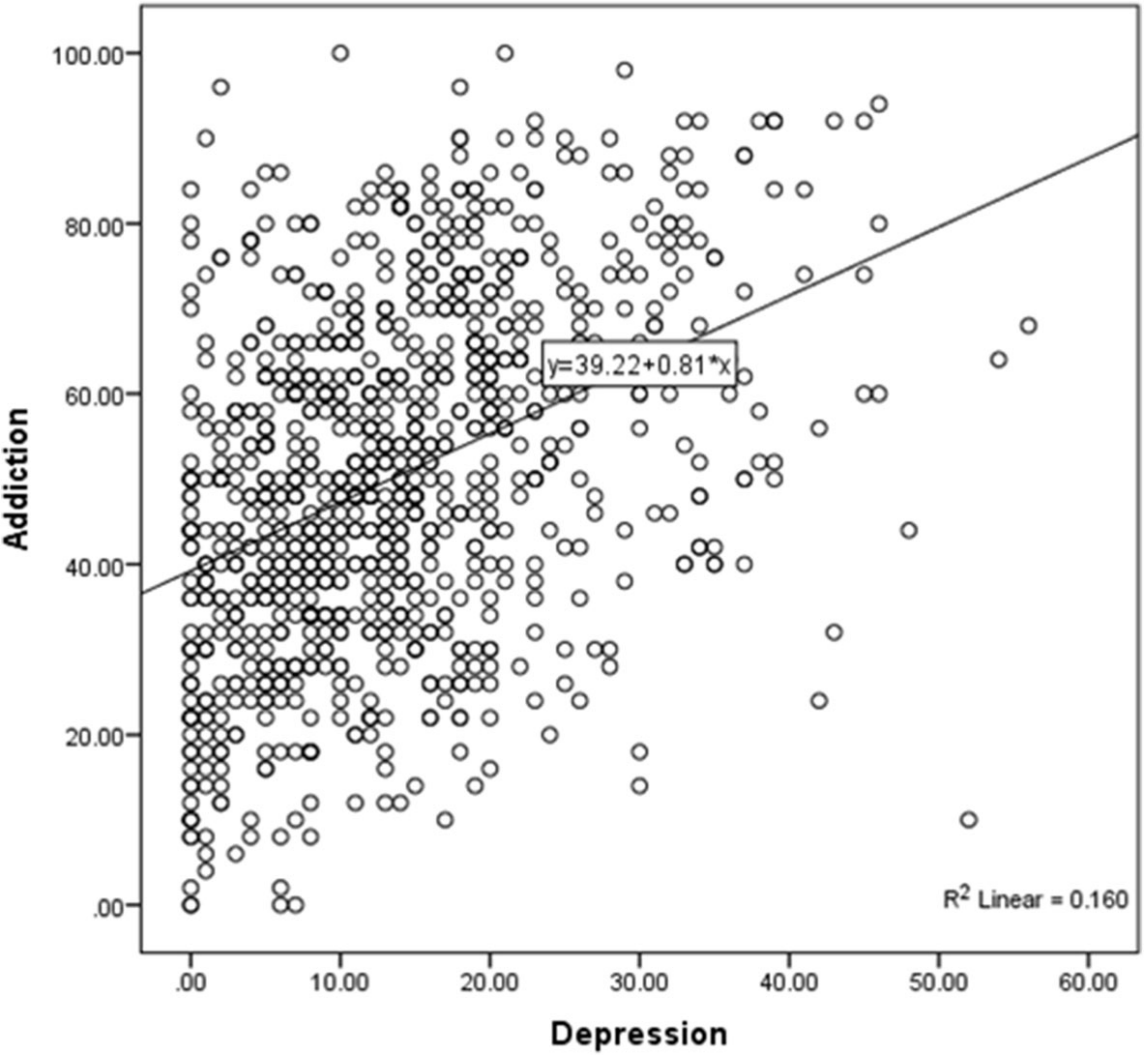
Relationship between smart phone addiction and depression scores

The overall percentage mean score (PMS) of Smartphone Addiction scale (SAS-SV) was 50.2 ± 20.25, while the mean score of Beck depression scale was 13.6 ± 10.0. The responses to Beck Depression Scale and Smartphone Addiction Scale individual statments are enlisted in suppliment Additional file 1: Tables S1 and Additional file 2: Tables S2 respectively.  Almost 19% can considered as non-addicted to smartphone usage (PMS = 0–30), 64% can considered as slightly addicted to smartphone usage (PMS = 31–70) and 17% as probably addicted to its usage (PMS = 71–100). In regards of depression, those classified as ups/downs to be normal were 39% (score 0–9), mild mood disturbance 25% (score 10–15), borderline clinical depression 12% (score 16–19), moderate depression 15% (score20–29), severe depression 7% (score 30–39) and extreme depression 1% (score ≥ 40). Qualitative characteristics of SAS-SV and Beck depression are illustrated in Figs. 1 and 2. A significant positive relation was observed between addiction and depression scores y = 39.2 ± 0.81×, *P* < 0.001, Fig. 3.

Initial bivariate analysis showed that the younger age group (PMS = 51.3 ± 20.5) had significantly the highest addiction scores compared to the older age group, *P* = 0.014. Smartphone users with school education (PMS = 15.7 ± 11.3), those with income <$1300 (PMS = 14.6 ± 10.2) and younger age group (PMS = 14.2 ± 10.2) were significantly more depressed than their counter groups (*P *= 0.003, *P* = 0.015 and *P* = 0.006 respectively), Table 2.

**Table 2:** Scores of smart phone addiction and beck depression scales across sample characteristics

	Smart phone addiction (0–100)	Beck depression (1–60)
	%mean score ± SD	Mean score ± SD
	50.2 ± 20.25	13.6 ± 10.0
Gender		
Male	50.9 ± 22.2	13.3 ± 10.5
Female	49.9 ± 19.2	13.8 ± 9.8
	*t* = 0.672, *P* = 0.502	*t* = − 0.806, *P* = 0.420
Age		
Young (18–35)	51.3 ± 20.5	14.2 ± 10.2
Middle (36–54)	48.9 ± 19.6	12.9 ± 9.5
Old (≥55)	40.2 ± 18.6	8.4 ± 8.96
	F = 4.301, df = 2, *P* = 0.014*	F = 5.167, df = 2, *P* = 0.006*
Educational level		
School	51.4 ± 20.9	15.7 ± 11.3
University	49.9 ± 20.1	13.2 ± 9.6
	*t* = 0.724, *P* = 0.395	*t* = 8.786, *P* = 0.003*
Income		
Less than $1300	50.2 ± 20.6	14.6 ± 10.2
Between $1300 & $3700	50.96 ± 19.8	13.4 ± 9.9
More than $3700	49.1 ± 20.3	12.1 ± 9.5
	F = 0.522, df = 2, *P* = 0.594	F = 4.221, df = 2, *P* = 0.015*

t = Student’s t-test; F = one-way ANOVA

*Abbreviations*: *ANOVA* analysis of variance, *SD* standard deviation, *df* degree of freedom

*P*-value = statistically significant at < 0.05, % = percentage

Two linear regression models were constructed and revealed that the factors that were significantly associated with higher smartphone addiction scores were: a) younger age (β = − 0.203, adj. *P* = 0.004) compared to older age smartphone users. Significant factors associated with higher depression scores were: a) School educated users (β = − 2.034, adj. *P* = 0.010) compared to university educated users; b) Users with higher smartphone addiction scores (β = 0.194, adj. *P* < 0.001) compared to users with less addiction scores, Table 3.

**Table 3:** Significant factors associated with higher smart phone addiction and depression scores

Variables	Smart phone addiction	Beck depression
	β (t)	β (t)
	Adj *P*-value	Adj *P*-value
Gender		
Female^a^	0.675 (0.461)	−0.355(−0.536)
Male^b^	*Adj P* = 0.645	*Adj P* = 0.592
Age	−0.203 (−2.892)	−0.050(−1.582)
(Years)	*Adj P* = 0.004*	*Adj P* = 0.114
Educational level		
School^a^	−1.878 (− 1.077)	− 2.034(− 2.579)
University^b^	*Adj P* = 0.282	*Adj P* = 0.010*
Income		
< $1300^a^	2.285 (1.398)	−0.781(−1.058)
≥ $1300^b^	*Adj P* = 0.162	*Adj P* = 0.291
Smart Phone Addiction		0.194 (13.119)
(%mean score)	–	*Adj P* < 0.001*
Constant	56.729 (22.33)	7.703 (5.414)
	*Adj P* < 0.001*	*Adj P* < 0.001*

t = Student’s t-test; ^**a**^**:** reference group,^b^: compared group

*Abbreviations*: *β* coefficient of determination, *Adj* adjusted

*Statistically significant at *P* < 0.05

## Discussion

The association between smartphone addiction and depression has been discussed in previous studies, but which preceded the other remains debatable. In this study, there was a strong positive relationship between smartphone addiction and depression. A systematic review of 23 peer reviewed papers reported that depression was consistently related to problematic smartphone usage [22]. Depression in Lebanese and Austrian university students was also significantly associated with smartphone addiction [12, 23] or problematic mobile phone usage [13] respectively. In fact, one of the predisposing factors of smart phone addiction is increased stress levels followed by a decrease in self-control, which eventually leads to the over usage of smartphones [24]. It is worth mentioning, that no relationship between smartphone ownership and symptoms of depression were reported by one study, which entails that addiction is related to self-control rather than the possession of the phone itself [25]. However, a negative association between smartphone addiction and depression was reported by another study [26]. Furthermore, another study stated that familial social support was a significant predictor of smartphone addiction, as this addiction increased as individuals’ familial support decreased [27]. There is no doubt that social relationships have been severely affected with the introduction of various social networks, and this phenomenon is commonly reported by the older generation who lived in the era before the introduction of smart phone technology. Unfortunately, there are gaps in literature on the probable association between smartphone addiction and social support [27]. Over usage of smartphones causes problems with attention and focusing, as these people are more likely to show more functional impairments that interfere with school, work and family life [28]. Young adults with personality type A who experience high stress level may lack positive stress coping mechanisms which makes them highly susceptible to smartphone addiction [8].

Observing the relationship between depression and smart phone addiction from an opposite perspective is also valid. Smartphone addiction can be a predisposing factor to depression, either indirectly or through a mediating effect. An association between smart phone addiction and altered lifestyle habits was found, with higher tendency among smart phone addicts to skip meals, to eat unhealthy diets, to gain weight, and to experience sleep disorders compared to less addicted smartphone users. These can be accounted as predisposing factors to depression [29]. Late night smartphone users indeed reported feeling tired (35.9%) and complained of less sleep quality (35.8%) [8]. This was confirmed by one Saudi study stating that higher problematic mobile usage scores were correlated with various unhealthy sleep hygiene, physical inactivity, dietary style and others [30]. One study reported that people with lower levels of self-perceived emotional or health conditions were more likely to exhibit an over usage of smartphones [31]. This indicates that these people are stuck in a vicious cycle. They are stressed from a certain emotional or health deficit, and then try to compensate or overcome this stress with on over usage of smart phones without realizing that this addiction itself leaves a negative impact on their social, emotional and physical wellbeing. On the other hand, an experimental study tried to determine the impact of not using a smart phone for 2 days. An alarming finding in this study was suggestive of an association between emotional dysregulation (suppression and decreased use of cognitive appraisal) and the psychopathology (depression, stress and anxiety) from social media loss [32]. Another study had claimed that neuroticism is linked in a chain-mediating effect with smartphone addiction and depression, all important variables that worsen the quality of life [33].

This study showed that the level of smart phone addiction was moderate, with 31.3% of subjects who can be accounted as addicts to smart phone usage. A meta-analysis paper claimed that the prevalence of smart phone addiction ranged between 39 and 40% [34]. One study proved that the fear of missing out was related to problematic smartphone usage [35]. Almost 60.9% of subjects in this study agreed to a certain degree that they constantly check their phones so as not to miss conversations between other people. Over usage of smartphones can actually impair the hand function by causing pain in the thumb and lowering the pinch strength [36]. Almost 33.8% of subjects in this study complained of physical pain in the wrist, back or neck. All these striking conclusions reveal that there is a clear association between smartphone usage addictions and various degrees of physical problems [37].

Younger individuals were more likely to be addicted than older individuals. A study from Turkey found that younger age groups had higher levels of smartphone overuse [12]. Generally, adolescents have a higher risk of smartphone addiction compared to adults, as they are more susceptible to accept new technologies than older generation groups [18]. Gender differences were not observed in this study, whether in relation to addiction or depression which was comparable to some studies in literature [19]. It was reported that males tend to use the internet mainly for online gaming, while females tend to use the internet for sending messages, chatting and blogging [26, 38]. Adults with higher educational level were less likely to have symptoms of depression, which was similar to results of a study conducted in 10 European countries [39]. A significant relationship between income and depression was noted as the people with lower income were more depressed. This was similar to findings of a Canadian study that found depression was less prevalent in those with a high income than those with a low income [23]. Younger adults seem to be more depressed than the older adults. This was consistent with findings in previous studies which can be attributed to the fact that younger adults are more susceptible to stressors such as social conflicts, marital relationships, raising children and others [40, 41].

### Limitations

Few limitations have been observed in this study, mainly due to the retrospective nature of the study design and the method of data collection. Recall bias is expected to be minimal as smartphone usage is experienced on daily bases and participants won’t have any difficulty recalling its influence on their various life aspects. Web-based surveys are usually linked to incentives, which make them prone to frauds. In this study, participation was both anonymous and free. The variable of marital status contracted errors during the extraction of data, and therefore was dropped out. There might be an association between marital status and the outcomes measured which could be investigated in future studies. Participation was not restricted to Saudi citizens and the job market in Saudi Arabia accommodates expatriate workers from all over the world, so the data collected is not only representative of the country itself. Generalizing the findings might not be self-limited to Saudi Arabia, as smart phone addiction is invading populations all over the world. Environmental and social characteristic of Saudi Arabia might be considered unique compared to other countries, but no studies have investigated the association between smart phone addiction and setting characteristics. The SAS-SV self-reported surveys grants researchers a reliable and valid assessment tool to identify addiction, yet a more accurate diagnosis is confirmed by a face to face assessment conducted by a clinical or behavioral expert. Depression is a sensitive outcome that cannot be linked exclusively to smartphone addiction; therefore the term “association” was used as a more conservative description of this relationship.

## Conclusions

The positive correlation between smartphone addiction and depression is alarming. Reasonable usage of smart phones is advised, especially among younger adults and less educated users who could be at higher risk of depression. The relationship between depression and smartphone addiction is an escalating global concern that necessitates future studies to investigate further on this aspect.

### Key points

- Depression is a mood disorder that is thought to be highly correlated with addiction to smart phone usage.
- An alarming positive correlation between addiction to smart phone usage and depression was found.
- Reasonable usage of smart phones is advised, especially among younger adults and less educated users who could be at higher risk of depression.

## Additional files

Additional file 1: **Table S1.** Responses to the statements in the Beck depression scale. (DOCX 23 kb)

Additional file 2: **Table S2.** Responses to the smart phone addiction scale. (DOCX 16 kb)

## Abbreviations

BDI II: Beck’s depression inventory
MNG-HA: Ministry of National Guard Health Affairs
MS: Mean score
PMS: Percentage mean score
SAS: Smartphone Addiction Scale
SAS-SV: Smartphone addiction scale-short version
WHO: World Health Organization
