# Supplementary material for: The relationship between addiction to smartphone usage and depression among adults: a cross sectional study
Source: BMC Psychiatry. 2018 May 25;18:148. doi: 10.1186/s12888-018-1745-4 (PMC5970452; doi:10.1186/s12888-018-1745-4)
Supplement: Supplementary file 1 — Table S1. Responses to the statements in the Beck depression scale. (DOCX 23 kb) [file 12888_2018_1745_MOESM1_ESM.docx]

Additional File 1: **Table S1** Responses to the statements in the Beck depression scale

| Depression scale responses | N (%) |
| --- | --- |
| 1. Sadness: |  |
| 1. I do not feel sad 2. I feel sad 3. I am sad all the time 4. I am so sad or unhappy that I can’t stand it | 528 (56.5)  348 (37.1)  23 (2.5)  36 (3.9) |
| 2. Pessimism: |  |
| 1. I am not discouraged about the future 2. I feel more discouraged about my future than I used to be 3. I do not expect things to work out for me 4. I feel my future is hopeless and will only get worse | 534 (57.1)  296 (31.7)  73 (7.8)  32 (3.4) |
| 3. Past Failure: |  |
| 1. I do not feel like a failure 2. I have failed more than I should have 3. As I look back, I see a lot of failures 4. I feel I am total failure as a person | 689 (73.7)  127 (13.6)  104 (11.1)  15 (1.6) |
| 4. Loss of Pleasure: |  |
| 1. I get as much pleasure as I ever did from the things I enjoy 2. I don’t enjoy things as much as I used to 3. I get very little pleasure from the things I used to enjoy 4. I can’t get any pleasure from the things I used to enjoy | 360 (38.5)  381 (40.7)  138(14.8)  56 (6.0) |
| 5. Guilty Feeling: |  |
| 1. I don’t feel particularly guilty 2. I feel guilty over many things I have done or should have done 3. I feel quite guilty most of the time 4. I feel guilty all the time | 220 (23.5)  585 (62.6)  103 (11.0)  27 (2.9) |
| 6. Punishment Feeling: |  |
| 1. I don’t feel I am being punished 2. I feel I may be punished 3. I expect to be punished 4. I feel I am being punished | 480 (51.3)  336 (36.0)  74 (7.9)  45 (4.8) |
| 7. Self-Dislike: |  |
| 0 I feel the same about myself as ever   1. I have lost confidence in myself 2. I am disappointed in myself 3. I dislike myself | 728 (77.9)  124 (13.2)  46 (4.9)  37 (4.0) |
| 8. Self-Criticalness: |  |
| 1. I don’t criticize or blame myself more than usual 2. I am more critical of myself then I used to be 3. I criticize myself for all of my faults 4. I blame myself for everything bad that happens | 450 (48.1)  190 (20.3)  169 (18.1)  126 (13.5) |
| 9. Suicidal Thoughts or Wishes: |  |
| 1. I don’t have any thoughts of killing myself 2. I have thoughts of killing myself, but I would not carry them out 3. I would like to kill myself 4. I would kill myself if I had the chance | 840 (89.8)  72 (7.7)  13 (1.4)  10 (1.1) |
| 10. Crying |  |
| 1. I don’t cry anymore than I used to 2. I cry more than I used to 3. I cry over every little thing 4. I feel like crying, but I can’t | 603 (64.5)  112 (12.0)  61 (6.5)  159 (17.0) |
| 11. Agitation: |  |
| 1. I am no more restless or wound up than usual 2. I feel more restless or wound up than usual 3. I am so restless or agitated that it’s hard to stay still 4. I am so restless or agitated that I have to keep moving or doing something | 614 (65.7)  230 (24.6)  39 (4.1)  52 (5.6) |
| 12. Loss of Interest: |  |
| 1. I have not lost interest in other people or activities 2. I am less interested in other people or things than before 3. I have lost most of my interest in other people or things 4. It’s hard to get interested in anything | 398 (42.6)  319 (34.1)  179 (19.1)  39 (4.2) |
| 13. Indecisiveness |  |
| 1. I make decisions about as well as ever 2. I find it more difficult to make decision than usual 3. I have much greater difficulty in making decisions than I used to be 4. I have trouble making any decisions | 501 (53.6)  287 (30.7)  59 (6.3)  88 (9.4) |
| 14. Worthlessness: |  |
| 1. I do not feel I am worthless 2. I don’t consider myself as worthwhile and useful as I used to 3. I feel more worthless as compared to other people 4. I feel utterly worthless | 701 (75.0)  120 (12.8)  101 (10.8)  13 (1.4) |
| 15. Loss of Energy: |  |
| 1. I have as much energy as ever 2. I have less energy than I used to have 3. I don’t have enough energy to do very much 4. I don’t have enough energy to do anything | 364 (38.9)  358 (38.3)  178 (19.0)  35 (3.7) |
| 16. Change in Sleep Pattern |  |
| 1. I have not experienced any change in my sleeping pattern 2. I sleep somewhat more/less than usual 3. I sleep a lot more/less than usual 4. I sleep most of the day/ I wake up 1-2 hours early and can’t get back to sleep | 275 (29.4)  442 (47.3)  116 (12.4)  102 (10.9) |
| 17. Irritability: |  |
| 1. I am no more irritable than usual 2. I am more irritable than usual 3. I am much more irritable than usual 4. I am irritable all the time | 458 (49.0)  346 (37.0)  63 (6.7)  68 (7.3) |
| 18. Change in Appetite: |  |
| 0 I have not experienced any change in my appetite   1. My appetite is somewhat less/greater than usual 2. My appetite is much less/greater than usual 3. I have no appetite at all/ I crave food all the time | 510 (54.5)  284 (30.4)  71 (7.6)  70 (7.5) |
| 19. Concentration Difficulty: |  |
| 0 I can concentrate as well as ever   1. I can’t concentrate as well as usual 2. It’s hard to keep my mind on anything for very long 3. I find I can’t concentrate on anything | 412 (44.1)  366 (39.1)  124 (13.3)  33 (3.5) |
| 20. Tiredness or Fatigue: |  |
| 1. I am no more tired or fatigued than usual 2. I get more tired or fatigued more easily than usual 3. I am too tired or fatigued to do a lot of things I used to do 4. I am too tired or fatigued to do most of the things I used to do | 366 (39.1)  368 (39.4)  127 (13.6)  74 (7.9) |

**n: frequency, %:percentage**
