# Supplementary material for: The relationship between addiction to smartphone usage and depression among adults: a cross sectional study
Source: BMC Psychiatry. 2018 May 25;18:148. doi: 10.1186/s12888-018-1745-4 (PMC5970452; doi:10.1186/s12888-018-1745-4)
Supplement: Supplementary file 2 — Table S2. Responses to the smart phone addiction scale. (DOCX 16 kb) [file 12888_2018_1745_MOESM2_ESM.docx]

Additional File 2: **Table S2**: Responses to the smart phone addiction scale

|  | Strongly disagree | Disagree | Weakly disagree | Weakly agree | Agree | Strongly agree |
| --- | --- | --- | --- | --- | --- | --- |
| 1.Missing planned work due to smartphone use. | 144 (15.4) | 176 (18.8) | 190 (20.4) | 275 (29.4) | 92 (9.8) | 58 (6.2) |
| 2.Having a hard time concentrating in class, while doing assignments, or while working. | 185 (19.8) | 223 (23.8) | 144 (15.4) | 218 (23.3) | 112 (12) | 53 (5.7) |
| 3. Feeling pain in the wrist or back of neck. | 135 (14.4) | 171 (18.3) | 105 (11.2) | 208 (22.2) | 183(19.6) | 133(14.2) |
| 4.Won’t be able to stand not having a smartphone | 47 (5.0) | 77 (8.2) | 110 (11.8) | 201 (21.5) | 185(19.8) | 315(33.7) |
| 5.Feeling impatient and fretful when not holding my smartphone | 95 (10.1) | 186 (19.9) | 149 (15.9) | 224 (24) | 154(16.5) | 127(13.6) |
| 6.Having my smartphone in my mind even when not using it | 152 (16.3) | 246(26.21) | 139 (14.9) | 181 (19.4) | 137(14.7) | 80 (8.5) |
| 7. I will never give up using my smartphone even when my daily life is already greatly affected by it. | 164 (17.5) | 212 (22.7) | 169 (18.1) | 172 (18.4) | 132(14.1) | 86 (9.2) |
| 8. Constantly checking my smartphone so as not to miss conversations between other people | 91 (9.7) | 153 (16.4) | 121 (12.9) | 214 (22.9) | 206 (22.0) | 150 (16.0) |
| 9. Using my smartphone longer than I had intended | 51 (5.5) | 111 (11.9) | 150 (16) | 203 (21.7) | 221(23.6) | 199(21.3) |
| 10. The people around me tell me that I use my smartphone too much. | 161 (17.2) | 243 (26.0) | 154 (16.5) | 154 (16.5) | 129(13.7) | 94 (10.1) |

**n: frequency, %:percentage**
